# CZ CELLxGENE Discover: a single-cell data platform for scalable exploration, analysis and modeling of aggregated data

**DOI:** 10.1093/nar/gkae1142

**Published:** 2024-11-28

**Authors:** CZI Cell Science Program, Shibla Abdulla, Brian Aevermann, Pedro Assis, Seve Badajoz, Sidney M Bell, Emanuele Bezzi, Batuhan Cakir, Jim Chaffer, Signe Chambers, J Michael Cherry, Tiffany Chi, Jennifer Chien, Leah Dorman, Pablo Garcia-Nieto, Nayib Gloria, Mim Hastie, Daniel Hegeman, Jason Hilton, Timmy Huang, Amanda Infeld, Ana-Maria Istrate, Ivana Jelic, Kuni Katsuya, Yang Joon Kim, Karen Liang, Mike Lin, Maximilian Lombardo, Bailey Marshall, Bruce Martin, Fran McDade, Colin Megill, Nikhil Patel, Alexander Predeus, Brian Raymor, Behnam Robatmili, Dave Rogers, Erica Rutherford, Dana Sadgat, Andrew Shin, Corinn Small, Trent Smith, Prathap Sridharan, Alexander Tarashansky, Norbert Tavares, Harley Thomas, Andrew Tolopko, Meghan Urisko, Joyce Yan, Garabet Yeretssian, Jennifer Zamanian, Arathi Mani, Jonah Cool, Ambrose Carr

**Affiliations:** Wellcome Sanger Institute, Wellcome Genome Campus, Hinxton CB10 1SA, UK; Chan Zuckerberg Initiative, 1180 Main Street, Redwood City, CA 94063, USA; Department of Genetics, Stanford University School of Medicine, 291 Campus Drive, Li Ka Shing Building, Stanford, CA 94305, USA; Chan Zuckerberg Initiative, 1180 Main Street, Redwood City, CA 94063, USA; Chan Zuckerberg Initiative, 1180 Main Street, Redwood City, CA 94063, USA; Chan Zuckerberg Initiative, 1180 Main Street, Redwood City, CA 94063, USA; Wellcome Sanger Institute, Wellcome Genome Campus, Hinxton CB10 1SA, UK; Department of Genetics, Stanford University School of Medicine, 291 Campus Drive, Li Ka Shing Building, Stanford, CA 94305, USA; Chan Zuckerberg Initiative, 1180 Main Street, Redwood City, CA 94063, USA; Department of Genetics, Stanford University School of Medicine, 291 Campus Drive, Li Ka Shing Building, Stanford, CA 94305, USA; Chan Zuckerberg Initiative, 1180 Main Street, Redwood City, CA 94063, USA; Department of Genetics, Stanford University School of Medicine, 291 Campus Drive, Li Ka Shing Building, Stanford, CA 94305, USA; Chan Zuckerberg, Biohub, SF, 499 Illinois St, San Francisco, CA 94158, USA; Chan Zuckerberg Initiative, 1180 Main Street, Redwood City, CA 94063, USA; Chan Zuckerberg Initiative, 1180 Main Street, Redwood City, CA 94063, USA; Clever Canary, 850 Front St. #1491, Santa Cruz, CA, USA; Chan Zuckerberg Initiative, 1180 Main Street, Redwood City, CA 94063, USA; Department of Genetics, Stanford University School of Medicine, 291 Campus Drive, Li Ka Shing Building, Stanford, CA 94305, USA; Chan Zuckerberg Initiative, 1180 Main Street, Redwood City, CA 94063, USA; Chan Zuckerberg Initiative, 1180 Main Street, Redwood City, CA 94063, USA; Chan Zuckerberg Initiative, 1180 Main Street, Redwood City, CA 94063, USA; Chan Zuckerberg Initiative, 1180 Main Street, Redwood City, CA 94063, USA; Chan Zuckerberg Initiative, 1180 Main Street, Redwood City, CA 94063, USA; Chan Zuckerberg, Biohub, SF, 499 Illinois St, San Francisco, CA 94158, USA; Chan Zuckerberg Initiative, 1180 Main Street, Redwood City, CA 94063, USA; Chan Zuckerberg Initiative, 1180 Main Street, Redwood City, CA 94063, USA; Chan Zuckerberg Initiative, 1180 Main Street, Redwood City, CA 94063, USA; Chan Zuckerberg Initiative, 1180 Main Street, Redwood City, CA 94063, USA; Chan Zuckerberg Initiative, 1180 Main Street, Redwood City, CA 94063, USA; Clever Canary, 850 Front St. #1491, Santa Cruz, CA, USA; Chan Zuckerberg Initiative, 1180 Main Street, Redwood City, CA 94063, USA; Chan Zuckerberg Initiative, 1180 Main Street, Redwood City, CA 94063, USA; Wellcome Sanger Institute, Wellcome Genome Campus, Hinxton CB10 1SA, UK; Chan Zuckerberg Initiative, 1180 Main Street, Redwood City, CA 94063, USA; Chan Zuckerberg Initiative, 1180 Main Street, Redwood City, CA 94063, USA; Clever Canary, 850 Front St. #1491, Santa Cruz, CA, USA; Department of Genetics, Stanford University School of Medicine, 291 Campus Drive, Li Ka Shing Building, Stanford, CA 94305, USA; Chan Zuckerberg Initiative, 1180 Main Street, Redwood City, CA 94063, USA; Chan Zuckerberg Initiative, 1180 Main Street, Redwood City, CA 94063, USA; Department of Genetics, Stanford University School of Medicine, 291 Campus Drive, Li Ka Shing Building, Stanford, CA 94305, USA; Chan Zuckerberg Initiative, 1180 Main Street, Redwood City, CA 94063, USA; Chan Zuckerberg Initiative, 1180 Main Street, Redwood City, CA 94063, USA; Chan Zuckerberg Initiative, 1180 Main Street, Redwood City, CA 94063, USA; Chan Zuckerberg Initiative, 1180 Main Street, Redwood City, CA 94063, USA; Chan Zuckerberg Initiative, 1180 Main Street, Redwood City, CA 94063, USA; Chan Zuckerberg Initiative, 1180 Main Street, Redwood City, CA 94063, USA; Chan Zuckerberg Initiative, 1180 Main Street, Redwood City, CA 94063, USA; Chan Zuckerberg Initiative, 1180 Main Street, Redwood City, CA 94063, USA; Chan Zuckerberg Initiative, 1180 Main Street, Redwood City, CA 94063, USA; Department of Genetics, Stanford University School of Medicine, 291 Campus Drive, Li Ka Shing Building, Stanford, CA 94305, USA; Chan Zuckerberg Initiative, 1180 Main Street, Redwood City, CA 94063, USA; Chan Zuckerberg Initiative, 1180 Main Street, Redwood City, CA 94063, USA; Chan Zuckerberg Initiative, 1180 Main Street, Redwood City, CA 94063, USA

## Abstract

Hundreds of millions of single cells have been analyzed using high-throughput transcriptomic methods. The cumulative knowledge within these datasets provides an exciting opportunity for unlocking insights into health and disease at the level of single cells. Meta-analyses that span diverse datasets building on recent advances in large language models and other machine-learning approaches pose exciting new directions to model and extract insight from single-cell data. Despite the promise of these and emerging analytical tools for analyzing large amounts of data, the sheer number of datasets, data models and accessibility remains a challenge. Here, we present CZ CELLxGENE Discover (cellxgene.cziscience.com), a data platform that provides curated and interoperable single-cell data. Available via a free-to-use online data portal, CZ CELLxGENE hosts a growing corpus of community-contributed data of over 93 million unique cells. Curated, standardized and associated with consistent cell-level metadata, this collection of single-cell transcriptomic data is the largest of its kind and growing rapidly via community contributions. A suite of tools and features enables accessibility and reusability of the data via both computational and visual interfaces to allow researchers to explore individual datasets, perform cross-corpus analysis, and run meta-analyses of tens of millions of cells across studies and tissues at the resolution of single cells.

## Graphical Abstract

**Graphical Abstract:**
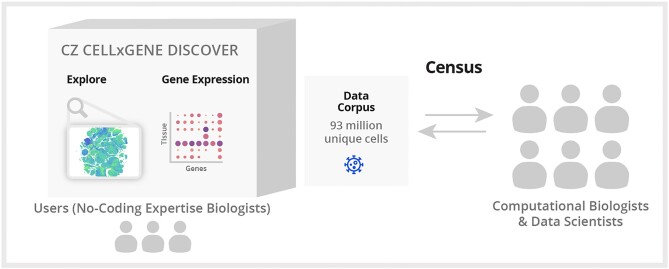

## Introduction

Cells have been the focus of scientific study for centuries and represent the fundamental unit of life (1). Biology and medicine have long histories of systematically observing, describing and classifying cells and the anatomical structures that they reside in using assorted methodologies. With each wave of technological innovation comes the discovery of new cell types or states, but also an equally important expansion of knowledge that defines the features of previously described cells in greater detail.

The goal of clarifying the molecular nature of cells has now come within reach due to advances in single-cell measurement technology and concerted community efforts (2,3). Over the past 5–10 years, research communities have mobilized for projects that span different tissues and organisms, deploying increasingly robust assays and pursuing large-scale characterization of cells that include the Human Cell Atlas (HCA) (2), Fly Cell Atlas (4), Tabula Sapiens (5), the Human BioMolecular Atlas Program (HuBMAP) (3) and many more. These communities have generated a wealth of data that describe how cells vary across organisms, tissues, sex, age and ancestries.

The unique characteristics of single-cell data, including the large number of individual measurements captured compared to other modalities (e.g. microarray, bulk RNA sequencing), impose requirements for curation and annotation. These challenges and requirements are not easily met using existing repositories. Several solutions arose due to these unique requirements, offering access to individual datasets and some larger specialized collections, such as the Lung Gene Expression Analysis portal (6), Allen Brain Map (7) and the Single-Cell Portal (8). Built-for-purpose portals enable rapid publication of studies and dissemination of unique biological features in specific datasets but lack the scalability and standardization needed for efficient meta-analysis. Even in the presence of such portals, efforts to explore or (re)analyze many datasets face a requirement to first standardize across individual data portals and resources, with only an estimated 25% of publicly available datasets providing the cell-level metadata needed for reuse (9). Data interoperability is a particularly important challenge for both individual users and the broader community to realize the promise of single-cell biology both now and in future applications that involve training models or assembling integrated references.

To address the need for standardized, interoperable and openly available single-cell matrices, we have developed Chan Zuckerberg CELLxGENE Discover (pronounced CZ CELL by GENE Discover). This rich platform pairs data and tools that enable scientists to find, download, explore, analyze and publish standardized single-cell datasets. The platform is open-source and free to use. Contributions of data are welcome from the scientific community and are not confined to a single consortium or funder. It serves as a centralized hub that promotes collaboration across researchers, labs and consortia. CZ CELLxGENE is differentiated from other single-cell data portals by its enforcement of a standardized schema for gene, cell, assay and donor metadata that evolves to address contributor requirements. This standardization provides the foundation for CZ CELLxGENE’s easy-to-use visual interfaces. Additionally, CZ CELLxGENE curators work jointly with data contributors on their submissions, rather than pull data from other public repositories or allow for automated submission. This collaborative approach ensures an accurate representation of the data, avoids different interpretations of the standards and results in richer metadata. CZ CELLxGENE serves only matrix-formatted data and metadata, which precludes the submission of raw sequence data. This allows for the open sharing of studies that may require controlled access for identifiable sequence data and enables increasingly equitable science for rapid insight into data without the immediate need for data reprocessing. CZ CELLxGENE Discover builds on the earlier work CZ CELLxGENE Annotate that enabled exploration, analysis and annotation of large-scale single-cell datasets but is optimized for dissemination and reanalysis (10).

CZ CELLxGENE is a suite of tools and features designed to address the challenges of compiling, curating and using data from across a diverse ecosystem. These challenges informed the development of scalable infrastructure, visualizations and interoperability of single-cell transcriptomic data. Specific features are built around datasets and collections that allow users to filter among data and download them along with standardized metadata in multiple formats. Features within CZ CELLxGENE, such as ‘Explorer and Gene Expression’, provide easy-to-use no-code web-based visualizations of data from a given dataset or collection and give users the ability to more dynamically explore questions across datasets. Finally, Census is an Application Programming Interface (API) and data object hosted by CZ CELLxGENE that enables efficient access and custom slices thereof for programmatic access and computational use cases.

**Figure 1.**
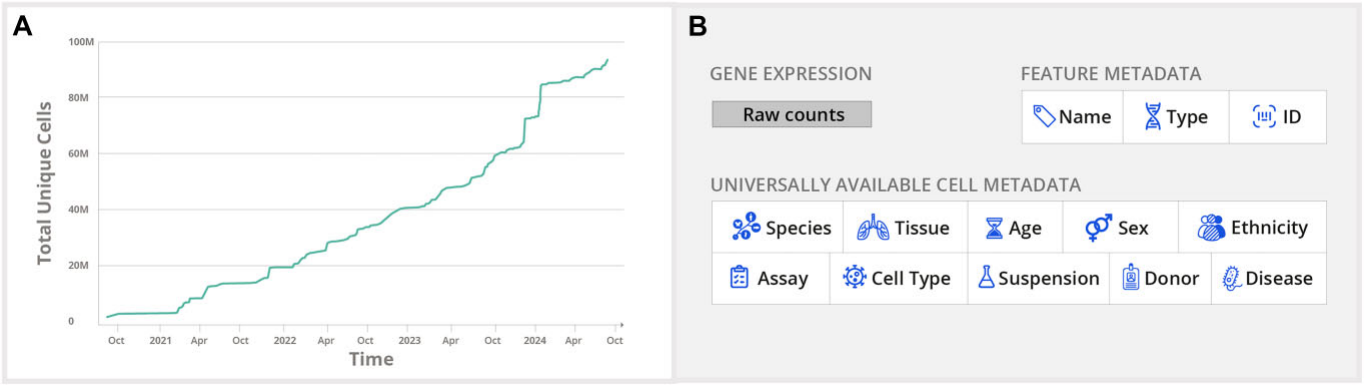
Description of CZ CELLxGENE schema and data curation efforts. (**A**) The total number of unique cells available on CZ CELLxGENE now surpasses 93 million cells. (**B**) All data on CZ CELLxGENE conforms to a standard metadata schema. The schema requires raw counts (e.g. mapped but unnormalized) as part of data submission. Required metadata covers 10 generally available categories that are completed for each sample and cell to increase reusability for downstream analyses. An additional metadata category, not shown in the figure above, is the is_primary_data field. This field is used to mark each observation as ‘primary’ exactly one time throughout the corpus so that cross-corpus aggregations can avoid redundant observations in their analysis.

Together, these features leverage both visual and computational interfaces to allow researchers in the single-cell community and beyond to rapidly explore individual datasets as analyzed and published by the original authors and to create corpus-wide views, summaries and meta-analyses of tens of millions of cells across studies. CZ CELLxGENE has been adopted as a primary data-sharing platform for small research labs and large consortia like BRAIN Initiative (11), Human Tumor Atlas Network (HTAN) and HCA. The data available in CZ CELLxGENE has continued to grow at a steady and impressive rate over the last 3 years (Figure1A). As of 1 October 2024, the platform hosts over 1550 datasets and 169.3 million cells (93.6 million unique cells), unlocking a new power for any researcher to ask and answer biological questions to clarify off-target effects of drugs across organs, identify unique marker genes or interrogate gene expression across cell types for all major human and mouse organs.

## Results

### CZ CELLxGENE data is universally standardized to a minimal data schema

Single-cell transcriptomic datasets from a large number of laboratories, institutions and consortia are available via websites and portals (12). Each portal serves the needs of its dataset, but typically fails to enable collective use. This presents a barrier to downstream use of the data and queries that run across many datasets. To address this challenge, we defined requirements and standards for a minimal schema that reflects widely used terms aimed at enabling corpus-wide searches, filtering of data and data analyses. Our driving motivation was to enable dynamic integration and queries of all the data or a subset of cells of interest to a biologist.

### A minimal cell-level schema

Metadata is critical for the reuse of data but often inconsistently captured or represented in single-cell datasets. We sought to define a schema that enabled data integration by defining a core set of metadata fields and ontologies. Integration enables the compilation of datasets into atlases and has become a core computational task within single-cell biology (13,14). It allows for the inference of multi-modal measurements, cell type prediction and cross-species analysis that underpins insights into a wide array of biological questions as well as the ambitions of community efforts to integrate individual datasets into references (15). Community input—including feedback from members of HTAN (16), HuBMAP (3) and HCA (4)—to develop standards in line with the key information frequently utilized by meta-analysis studies to properly integrate data or to identify biological variables correlated with gene expression (17–19). To avoid deterring or inhibiting data submission and adoption, we limit the schema to 11 required fields considered most valuable for data integration and reuse. The resulting requirements were encoded into a minimal, versioned schema that all submitted data must adhere to and be validated against during the submission process (Figure 1B, Supplementary Table S1).

The required fields represent attributes that are often variable within or across studies and are often identified as strong covariates correlated with gene expression variation within cells (20,21). Similar to previous experimental data coordination efforts, established ontologies and other community resources are used for standardization wherever possible for consistency and to improve the filtering capabilities of datasets (Supplementary Table S2) (22–24).

To fully capture gene count information for each dataset, a layer of raw data, meaning non-normalized mapped reads, is required for submission of data from all transcriptomic assays to fulfill common computational reuse cases (25–27). The corpus-wide availability of raw counts enhances data accessibility by openly serving reusable data products for studies with controlled access sequence data and prevents computational resources from being used towards re-alignment for cases where the original alignment will suffice.

CZ CELLxGENE does not recluster or perform analyses on individual datasets. As a result, at least one two-dimensional embedding, such as Uniform Manifold Approximation and Projection (UMAP), t-Distributed Stochastic Neighbor Embedding (tSNE), Principal Component Analysis (PCA), etc., is required to facilitate dataset visualization within the Explorer interface (see the ‘Scalable tools allow biologists to explore, query and analyze CZ CELLxGENE data’ section). Importantly, CZ CELLxGENE allows for multiple embeddings, enabling the sharing and exploration of diverse data representations.

Metadata for a given dataset is not constrained to CZ CELLxGENE’s minimal schema; it is extensible and allows for the submission of additional metadata that contributors consider valuable. In the case of coordinated efforts or consortia, the system fully supports additional fields that have been standardized across studies to encourage and enable meta-analysis. The full schema specifications have been adopted by multiple consortia, including the HCA, BRAIN Initiative and Kidney Precision Medicine Project (28), and are available to the research community to support interoperability with current and future efforts (Supplementary Table S1).

### Schema evolution

Schema evolution is anticipated given the advancement in data generation techniques and data analysis technology, especially in a rapidly advancing scientific field like single-cell biology. We designed the minimal schema to be supportive of changes and review the data corpus every 6 months for opportunities to make it increasingly comprehensive and standardized. With each schema update, previously submitted data are migrated to meet the new schema, even if that requires re-curation (see the ‘Materials and methods’ section). These data migrations ensure that all datasets are consistently described by the most current standards at all times, and users will have consistent filtering and integration experiences independent of when a given dataset was submitted. The full CZ CELLxGENE schema changelog is available (Supplementary Table S1).

### Submission workflow

CZ CELLxGENE welcomes data contributions meeting the current submission criteria (Supplementary Table S1) from any individual contributor, laboratory or institution. Upon request from data contributors via email to cellxgene@chanzuckerberg.com, a dedicated curation team creates a new private Collection, defined as a group of datasets that are part of a study or publication, with a contributor-provided title, description, contact, associated consortia or projects, as well as any external URLs (Supplementary Table S2). To enable collaboration and journal reviewer access without registration, the curation team provides the contributor with a URL to their new Collection. This URL is permanent and will not change when the Collection is made public such that contributors do not need to update their manuscript or other text where the URL is referenced. This URL is also obscure such that until the Collection is made public, the URL is only viewable by the contributor and anyone they share the URL with. CZ CELLxGENE does not include any alignment, cluster or annotation pipeline so all requirements, including cell population labels, must be provided by the contributor based on their own analysis. Once a dataset fulfills the schema requirements, it is uploaded to the Collection as an H5AD file in AnnData format (29). Upon upload, the AnnData object is updated with human-readable gene symbols and ontology labels based on the submitted identifiers. A Seurat object is then created from the updated AnnData using sceasy (30). Both formats are made available to consumers for download to enable reuse in a variety of downstream single-cell analysis toolchains. In addition, an internal format for visualization in Explorer is produced. Gene symbols and ontology labels are mapped consistently throughout the data corpus via a specific ontology release version defined in the schema for each community resource.

Additional details on the data submission process are available on the CZ CELLxGENE documentation pages (Supplementary Table S1).

### Community resource for standardized cell resolved measurements

**Figure 2.**
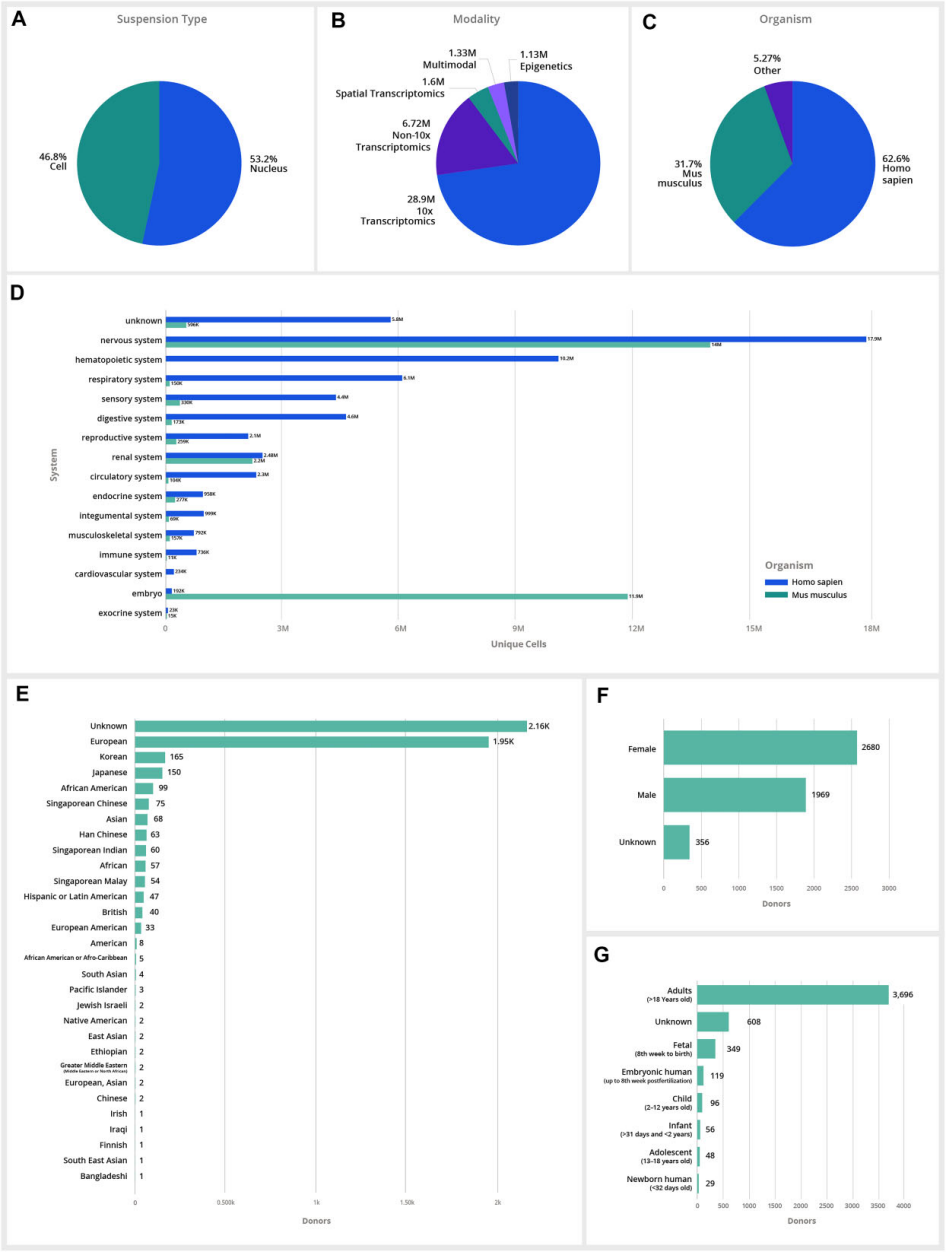
CZ CELLxGENE data corpus across various metadata categories. (**A–C**) A breakdown of total unique cells in CZ CELLxGENE by suspension type, modality and organisms showing the majority of data available in CZ CELLxGENE is generated from human and mouse tissue using 10X Genomics transcriptomic assays. Data is available across additional modalities (e.g. non-10× transcriptomic assays, spatial transcriptomics, epigenomic and multimodal data types, are supported if they meet the minimal schema requirements) and species (e.g. *Macaca mulatta, Pan troglodytes* and others) are supported if they meet the minimal schema requirements. **(D)** A breakdown of the total unique cells across all major organ systems for both mouse and human available in CZ CELLxGENE. (**E–G**) A summary of human data across self-reported ethnicity, developmental stage and sex.

CZ CELLxGENE hosts the largest curated collection of publicly available single-cell data, with data obtained from both single cells and single nuclei across 449 tissues and 40 unique assay types transcriptomics (Figure 2A–C). The CZ CELLxGENE schema and underlying architecture have been designed for extensibility to new modalities and growth of the data corpus. Current data is primarily single-cell transcriptomic, although CZ CELLxGENE accepts additional modalities to support the growing number of studies that incorporate a multitude of assays and co-assays (Figure 2B). New data will require ongoing evolution of refined human cell types and tissues in a standardized, structured way by leveraging and contributing to the Cell Ontology (31), Human Ancestry Ontology (32) and UBERON (33).

Of the 93+ million unique cells available in the data corpus, 63% of the total cells in the corpus are human, 32% are mouse and <5% are from other species (Figure 2C). Of the human data, ∼62% is defined as coming from healthy donors, and the remaining 38% span 132 unique diseases. While CZ CELLxGENE data corpus remains the largest publicly available single-cell data resource, significant data deficiencies exist in many tissues and systems across both mouse and human (Figure 2D). When considering the comprehensiveness of human data available in the data corpus, samples from female tissue donors are slightly more numerous across the data corpus, and diverse ethnicities and age groups are grossly underrepresented, with the majority of data coming from adults of European or Unknown ethnicity (Figure 2E–G). It should be noted that CZ CELLxGENE’s data schema requirements provide the ability to assess the comprehensiveness and deficits of CZ CELLxGENE data corpus across metadata categories.

### Navigating the CZ CELLxGENE data corpus

CZ CELLxGENE provides two main ways to interactively browse data: the Collections and Datasets pages. The Collections page allows users to view all Collections, defined as thematic groups of data, typically grouped by a publication, in the data corpus and filter by metadata categories. If a Collection has a publication DOI, then its author information, journal and publication date are retrieved from the Crossref service to filter by Publication Date or Publication. Each collection also provides contributor and provenance information including contact, narrative description and an association with a consortium if appropriate. From the Collections page, one can view high-level attributes of the data contained within each collection [e.g. organism (s), tissue (s) and disease (s)], and a short citation of the associated publication, if one is present.

The Datasets page allows for viewing and filtering all datasets in the data corpus by the same metadata as the Collections page, plus cell and gene counts, and provides access to the downloadable files and Explorer visualization for each dataset. Clicking on a Collection title from either search page will direct users to the corresponding Collection page where one can view the Collection information and a Dataset table that provides summary metadata for each Dataset in the Collection as well as file and visualization access (Supplementary Figure S1).

### Scalable tools allow biologists to explore, query and analyze CZ CELLxGENE data

A universal view of atlas data represents an important next step to encourage greater insight from the immediate users of the data and wider sections of the scientific community. The preponderance of tissue and multi-organ atlases provide ample data but raise a myriad of challenges related to interoperability, data format and the ability of computational tools to quickly query 10s or 100s of millions of cells. CZ CELLxGENE addresses several challenges associated with data aggregation and standardization to enable researchers with wide ranges of expertise to visualize, explore, access and reuse single-cell data. Currently, we provide three main tools: Explorer, Gene Expression and Census, all of which utilize standardized data to lower the bar for integration as well as allow immediate exploration of biological insights.

### Explorer allows interactive exploration and analysis of individual single-cell datasets up to 4 M cells

**Figure 3.**
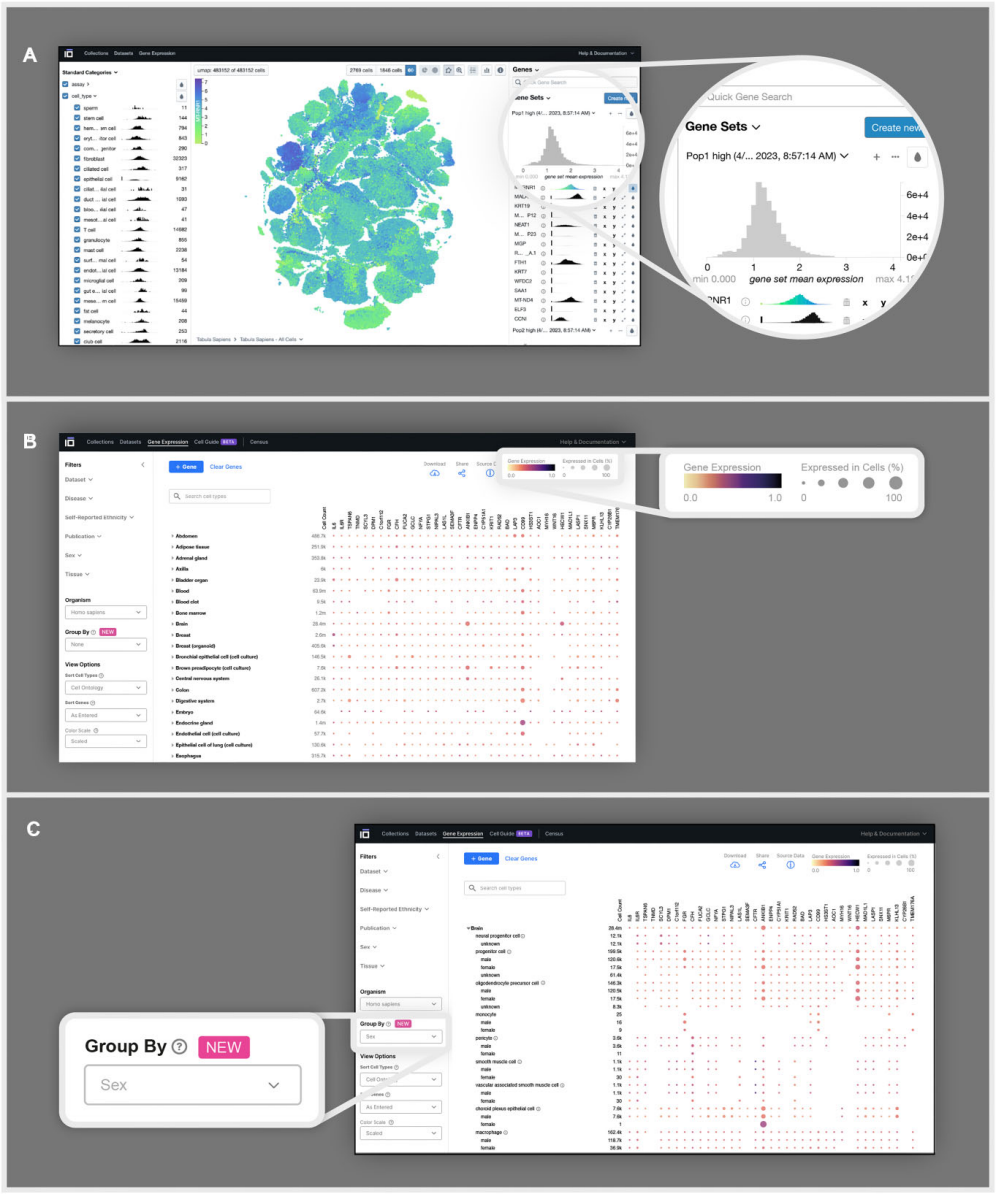
CZ CELLxGENE features Explorer and Gene expression enable interactive analysis of single-cell datasets. (**A**) A UMAP of all 483 152 cells in the Tabula Sapiens dataset available on Explorer visualized by the expression of MT-RNR1, a gene that encodes for an ribosomal RNA responsible for regulating insulin sensitivity and metabolic homeostasis (36). UMAPs can be visualized based on metadata categories, including cell type and other metadata categories, or by the expression of one or multiple genes, as shown above. (**B**) A heatmap generated using Gene Expression visualizing the mean gene expression of specific genes across and within all tissues and cell types present in the data corpus, where the quantity of cells used for calculating the mean gene expression is indicated in the leftmost column of the heatmap under ‘cell count.’ The gene expression is displayed using two visual elements: color, representing the mean gene expression and size, signifying the proportion of cells in each cell type or tissue expressing the respective gene. (**C**) A heatmap generated using the Group By feature demonstrates the variation in gene expression among different cell types according to sex. Group By allows researchers to group mean gene expression values by specific metadata values, including, sex, disease and ethnicity.

The majority of single-cell visualization portals rely on precomputed values that limit interactive features on datasets and generally are tuned to host datasets of 250–500k cells (30). Portals have struggled to keep up with the growth of dataset size, now reaching the order of millions of cells per study (Figure 3A) (34,35). As the field scales, and perhaps importantly, as more integrated datasets become available, it is critical to have the ability to dynamically visualize and perform basic analysis functions on millions of cells. Explorer, a feature of CZ CELLxGENE, is a visualization platform that allows researchers to dynamically explore, compute and query individual datasets for up to 4.3 million cells in <1 min (see the ‘Materials and methods’ section).

With Explorer, researchers can explore gene expression by visualizing individual genes or groups of genes (Figure 3A, inset); performing differential gene expression (Welch’s *t*-test); identifying marker genes; and visualizing continuous (e.g. UMI count) and categorical (e.g. cell type) cell metadata. Explorer offers a dynamic interface that allows users to rapidly explore co-variation and trends that are not typically captured or presented in pre-computed interfaces. Users can facet these visualizations based on gene expression, metadata and the embedding itself. The combinatorial nature of these affordances facilitates arbitrary comparisons through cross-filtering, subsetting and coloring subpopulations of the data (Figure 3A).

### Gene Expression allows gene expression queries across the corpus of data

An immediate opportunity of single-cell atlases is to offer researchers the ability to interrogate the expression of genes across cell types in a specific biological context, such as tissue, disease, ancestry, sex and developmental stage. To meet this need, we developed Gene Expression, an interactive tool with an intuitive visual interface to explore gene expression across all RNA datasets hosted on CZ CELLxGENE that meet assay and quality criteria (see the ‘Materials and methods’ section).

Gene Expression displays heat map visualization of messenger RNA levels across cell types and tissues. For any given cell type and gene, the dot color represents the average gene expression of all cells annotated accordingly. The size of each dot represents the percentage of cells within the cell type that express that gene (Figure 3B). This visualization reveals high-level differences in expression patterns across cell types and tissues. The combination of these metrics in a grid of [*cell types* by *genes*] allows researchers to make qualitative assessments of gene expression between user-defined subsets of cell types and tissues. Researchers can filter by tissue, publication, sex, self-reported ethnicity and disease to tailor their results to a subset of the data they are interested in or deem most relevant. The Group By functionality reports gene expression for cell types stratified by the selected category (i.e. sex, self-reported ethnicity and disease), further enabling researchers to identify gene expression that define and differentiate cell types in different biological contexts (Figure 3C).

### Gene Expression provides an aggregated view of cell types’ transcriptional states and marker genes

Gene Expression's underlying data is a concatenation of single-cell transcriptomic sequencing data, normalized with log pseudocounts and filtered for quality (see the ‘Materials and methods’ section). We then (i) aggregate this normalized data and (ii) generate marker genes.

### Aggregation partially mitigates batch effects

Single-cell transcriptomic data suffers from batch effects which distort biological signal when comparing between datasets. Despite many advances in methods development for data integration, the current state-of-the-art methods produce output in a lower-dimensional space than the original count matrix, making them unsuitable for our application. Thus, our simple pre-processing pipeline uses log transformed pseudocounts to normalize each cell’s gene expression data, but this does not aim to eliminate batch effects between datasets. However, we wanted to understand the extent to which simply *aggregating* (averaging) these normalized values across many cells and datasets is able to partially mitigate these batch effects.

**Figure 4.**
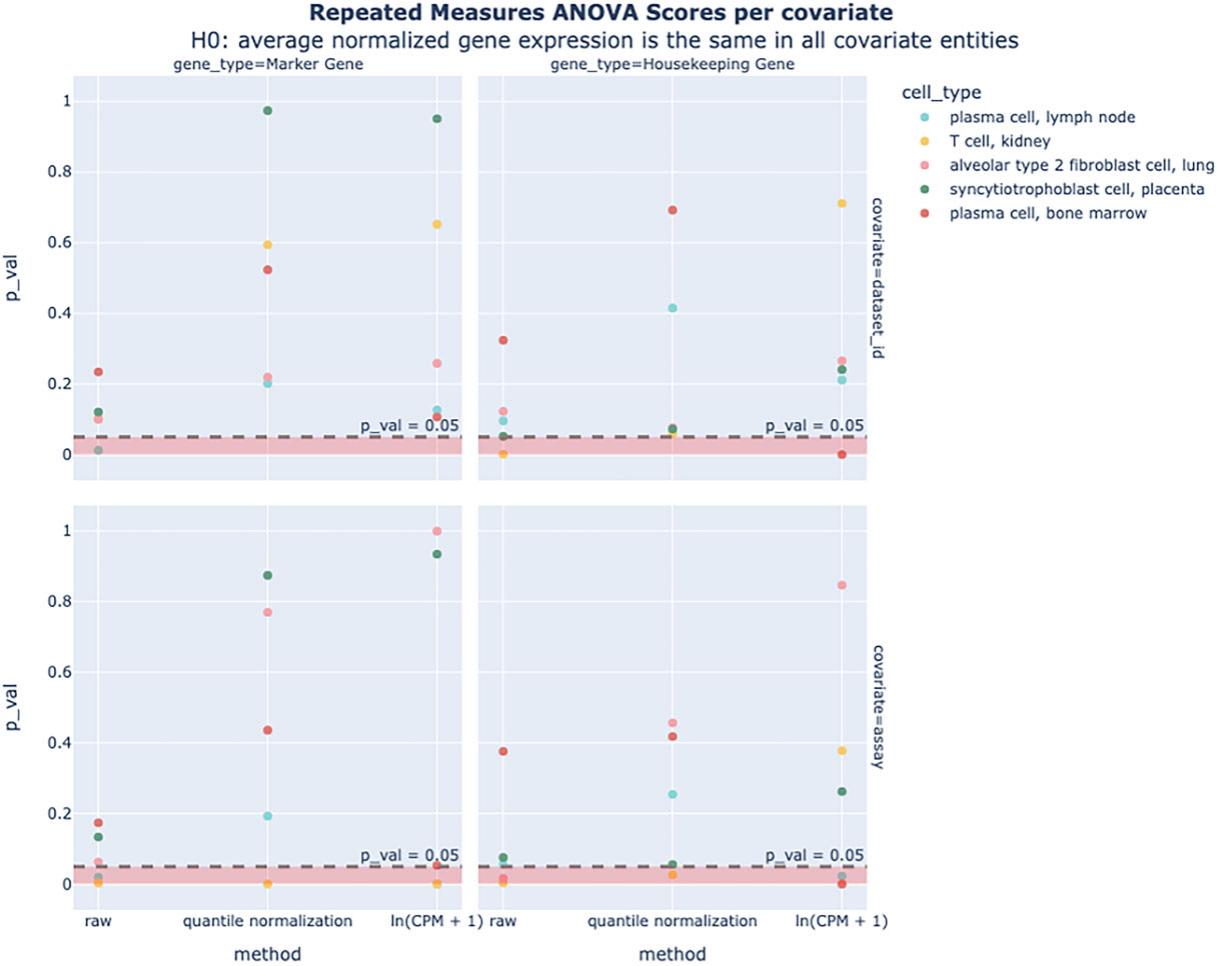
ANOVA on average normalized gene expression values. Results for one-way repeated measures ANOVA scores conducted on marker and housekeeping genes in five different cell types. Results show that for most cell types, we do not have sufficient evidence to say that there is a statistically significant difference between the average normalized gene expression values among covariate values [9/10 and 6/10 *P* values for ln (CPM + 1) for dataset_id and assay *P*_val_ = 0.05, aggregated over marker and housekeeping genes].

To directly quantify this, we used a repeated measures Analysis of Variance (ANOVA) analysis to assess the extent to which average gene expression vectors of several cell types vary between datasets and assays. For both marker genes and housekeeping genes, our results show that for most cell types, we do not have sufficient evidence to say that there are significant differences between the average gene expression values across covariates (Figure 4). We also found that log normalization further reduces batch effects (Supplementary Figure S2). This suggests that batch effects are at least partially mitigated by aggregation and log normalization compared to raw counts; however, users should be advised that the displayed values may still be affected by batch effects, especially for rare cell types.

### Gene Expression provides computationally derived marker genes for most cell types

**Figure 5.**
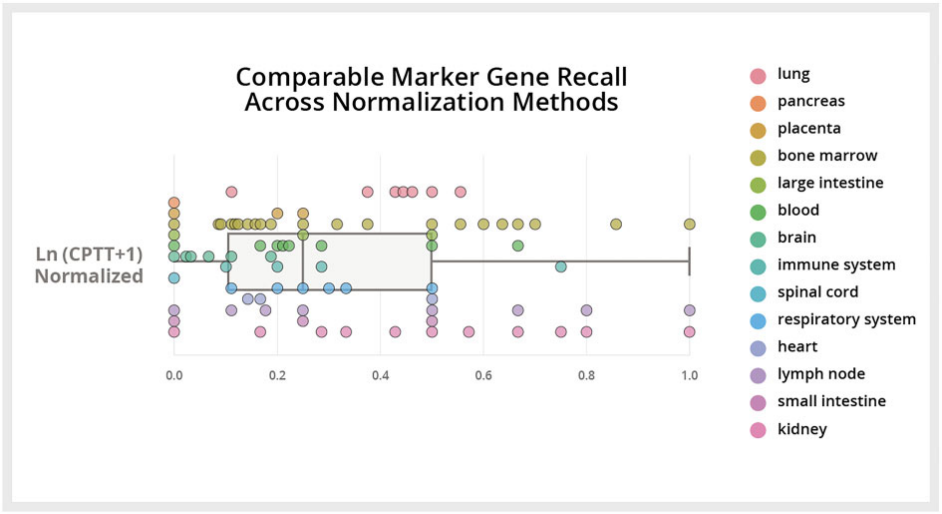
Recall of marker genes: comparison between raw counts, quantile normalization and log transform normalization. Each point represents the sensitivity of marker gene recall for a specific cell type and tissue, as compared to canonical marker genes from HuBMAP.

Marker genes are widely used in single-cell analysis but are inconsistently annotated for many cell types, especially across different tissue contexts. Gene Expression provides computationally derived marker genes for nearly every cell type, specific to each tissue context. To evaluate these, we estimated the sensitivity of a *t*-test (which is the basis of the Gene Expression marker gene pipeline; see the ‘Materials and methods’ section) in recalling marker genes from HuBMAP for 127 cell types from 14 tissues (37). Cell type selection was based on data availability; where two cell types were colinear in the Cell Ontology, we removed the more basal type. We found an average recall of 33% (Figure 5). The probability of observing this level of sensitivity by chance for each cell type ranges from *P* = 0.00–0.04; the moderately low sensitivity values are likely influenced by the fact that the HuBMAP dataset we use as our ‘ground truth’ includes annotations from a variety of different assay types and are quite noisy. Marker genes can be identified and visualized in the Gene Expression interface (Supplementary Figure S3).

### Census provides efficient programmatic access to CZ CELLxGENE data for flexible exploration and reanalysis

A central goal of the CZ CELLxGENE effort is to ensure robust programmatic access to a corpus of interoperable data for reuse and atlas-level analysis and modeling. To support this, we developed a global view of the data, termed Census, as a service composed of a cloud-hosted data object hosted at Amazon's Open Data registry along with Python and R packages for efficient computing and access to non-spatial transcriptomic data available in CZ CELLxGENE (see the ‘Materials and methods’ section).

Many tools that are widely used for analysis, or even meta-analysis, are not designed to operate on datasets of this scale (38). There are a myriad of existing computational analytical tools and methods available to the community (39–42), many of which have made significant advancements towards increasing scalability around data access and analysis. For example, for R users, Seurat V5 (43) introduced a new paradigm to perform memory- and compute-costly processes on subsets of a dataset, while performing light-weight processes on full datasets. Similarly, for Python users, AnnData introduced on-disk representations of data while incorporating state-of-the-art parallel computing via Zarr (44) and Dask (45), respectively. While these tools are increasing the scale to which computational biologists can access and analyze data, many of these solutions are still limited to only millions of cells and more importantly, lack of interoperability between R and Python. To fill this gap, and in particular, to allow interoperable and scalable data access beyond 10s and 100s of millions of cells, we co-developed TileDB-SOMA (Supplementary Table S1, see the ‘Materials and methods’ section). This API is intentionally designed for data access at scale while maintaining interoperability with the existing computational tools in the single-cell ecosystem. TileDB-SOMA was then used to build Census – a large data object hosted in the cloud with initial data covering all RNA non-spatial transcriptomic data from CZ CELLxGENE.

**Figure 6.**
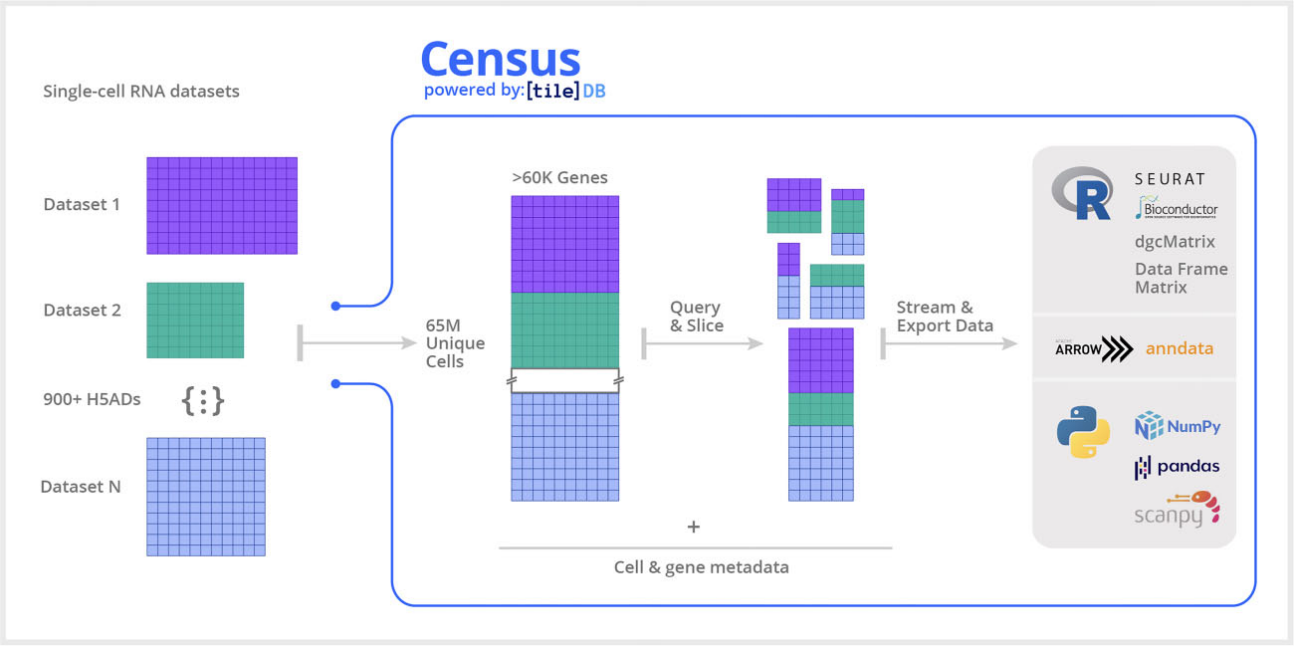
Overview of Census framework. Census is built upon the TileDB-SOMA framework to enable computational scientists to execute complex and specific queries across over 65 million cell measurements compiled from 900+ datasets spanning human and mouse organs available in CZ CELLxGENE using Census. Leveraging out-of-core processing, SOMA provides the API and data model to facilitate the storage, retrieval and analysis of datasets exceeding memory capacity. The standardized schema required by the data portal enables users to effortlessly query and export any segment of the extensive 65+ million cell dataset for in-depth analysis using Python and R.

Through its cloud-based platform, Census offers efficient, low-latency access for larger-than-memory slices of CZ CELLxGENE data, which users can access through Python and R APIs by performing queries based on cell or gene metadata (Figure 6). Census is interoperable across existing single-cell toolkits as query results can be exported to Seurat, AnnData or SingleCellExperiment objects. Notably, Census offers interoperability with basic language structures: from Python, it can export data to PyArrow objects (46), SciPy sparse matrices (47), NumPy arrays (48) and Pandas data frames (49). From R, Census can export data to R Arrow objects, sparse matrices (via the Matrix package) and standard data frames and dense matrices.

Importantly, TileDB-SOMA allows Census to interact with CZ CELLxGENE data in an out-of-core fashion via its iterable-based streaming capabilities (see the ‘Materials and methods’ section). Concretely, this means that data can be queried, processed and analyzed in data chunks of fixed size. Algorithms can then be adapted to work on an incremental basis; particularly any computation that can operate independently on per-cell or per-gene data can be easily implemented in this manner. Computations that require random access to the full query result or multiple passes through the data can be redesigned as an on-line algorithm (50). In Census, we have already deployed out-of-core implementations for some commonly used computations in single-cell, including incremental mean and variance calculation, and the ability to identify highly variable genes, all of which can now be executed in a regular 8 GB memory laptop across 65 million cells and >60 000 genes. These out-of-core functionalities are available in the Census Python package (Supplementary Table S1).

Modeling of single-cell data at scale is an important aspect of Census, as well as the broader single-cell field, given its demonstrated uses for *in silico* experimentation (51,52), data integration and annotation (53,54), cell state prediction (55,56) and clinical applications (57–59). PyTorch (60) is one of the most popular machine learning frameworks in single-cell, with notable models built using the PyTorch library including scvi-tools, Geneformer (61) and scGPT (62). To allow existing and new machine learning models to be trained on Census-scale data, we implemented PyTorch iterable data loaders that work natively with Census via TileDB-SOMA. Leveraging the modular design of the PyTorch libraries, the Census data loaders can be easily utilized in new and existing model training pipelines, allowing models to be trained on Census-scale data using readily available computing resources.

Initial datasets included in Census were generated using non-spatial RNA technologies, contain cells from human or mouse, provide raw counts and utilize only standardized cell and gene metadata as described in the CZ CELLxGENE dataset schema description above. New data is regularly added to Census and more details on current inclusion criteria can be found within Census Documentation (Supplementary Table S1).

## Discussion

CZ CELLxGENE provides an interoperable and dynamic community resource that supports a diversity of biological and computational applications. Diverse use cases are enabled by a uniquely large, multi-organ and consistently curated data resource. Within the CZ CELLxGENE platform, this data resource is served in a variety of ways that enable rapid and dynamic exploration at the dataset and corpus level. Examples of use include clarifying the mechanism by which aging can drive B-cell lymphoma (63) and identified signaling gene sets involved in small cell lung cancer (64). The standardized data from CZ CELLxGENE has enabled the development of new computational tools including UniCell: Deconvolve Base (UCDBase), a pre-trained, interpretable, deep learning model to deconvolve cell type fractions and predict cell identity across transcriptomic datasets (65); scTab, a cell type prediction model (66); and CSeQTL, a tool for mapping cell type-specific gene expression quantitative trait loci (67).

Compilation and standardization of single-cell transcriptomic datasets reduces barriers to meta analysis. CZ CELLxGENE’s cell-level schema is important for this work as it enables corpus-wide analysis but also user-defined subsetting of the data to related collections of cells that span multiple datasets. For example, we developed the Gene Expression interface to enable researchers to quickly identify computationally computed marker genes for over 900 cell types and begin to understand the expression of a gene of interest. Gene Expression does not fully correct for batch effects but is well suited for many use cases. Normalization with log-transformed scaled pseudocounts meets the scalability requirements for processing large datasets efficiently, allowing for the iterative addition of new cells without necessitating a full re-run of normalization processes. It also successfully stabilizes variance for visualization purposes while providing a complete count matrix of expression values (rather than low-dimensional representations). These findings collectively support the suitability of log transform normalization for the Gene Expression tool so long as users are aware of its shortcomings.

Deep learning and generative modeling provide an exciting opportunity to address many of these challenges (61,62). A recent demonstration of the power of CZ CELLxGENE data is on its application to train scGPT, a generative pre-trained model, on 33 M cells, which the authors used for cell-type annotation, multi-batch integration, multi-omic integration, genetic perturbation prediction and gene network inference. Other forms of global representations that utilize CZ CELLxGENE are delivering value for scientists seeking to contextualize their data against large reference collections (68). Currently, models trained on CZ CELLxGENE Census data are available, and regularly retrained as the data corpus grows, for downstream use. However, users do not have the ability to directly interact with models via the hosted web interface. Workflows, such as cell type prediction, projecting a new dataset onto the reference corpus and alignment of unanalyzed modalities, still require downloading an embedding and working outside of the platform. Future work is exploring opportunities to expand access to models and provide a richer exploration experience of datasets within CZ CELLxGENE. Given the potential utility that models provide across many tasks, we envision future work that will enrich the experience of exploring datasets hosted in CZ CELLxGENE but also the potential of enabling users to explore newly generated data not yet hosted in the platform.

As the single-cell biology field continues to evolve, it is critical that data portals and resources provide a consistent, reliable framework, while also adapting to the emerging technologies and the trajectory of the field. To this end, ongoing work includes updating all CZ CELLxGENE features (Gene Expression, Marker Genes, Census) to present data that is derived from the same versioned snapshot of Portal data. Additionally, there is a need to further validate the normalization methods used across the corpus. Importantly, the field has acknowledged the importance of spatial information in the context of single-cell biology (69,70), and ongoing work in both CZ CELLxGENE Explorer as well as Census seeks to support these efforts and technologies. Finally, we recognize the ongoing importance of new modalities beyond dissociated RNA and have begun to provide support for a growing array of data modalities.

In summary, the CZ CELLxGENE platform not only enables data sharing and data reuse through its standardized schema and curation pipeline but also allows researchers across expertise to access and visualize the largest single-cell dataset to date, with over 90 million cells across 449 tissues. Critically, the Census has unlocked a new potential for biological insights at scale and provides a low-friction bridge between single-cell biology and the machine learning community. Given the rapid advances in the field of Machine Learning and Artificial Intelligence, we anticipate that many additional applications of large language models and modeling approaches will be powered by Census and the CZ CELLxGENE suite of tools while encouraging deep biological studies that leverage insight from millions of cells resolved measurements presented in an easy-to-use platform.

## Materials and methods

### Performant visualization and analysis for up to 4 million cells via explorer

To achieve unparalleled efficiency, Explorer is engineered to incorporate numerous state-of-the-art tools and protocols. At the heart of its memory efficiency lies the use of TileDB Embedded (https://tiledb.com/products/tiledb-embedded/) as its data storage engine, a powerful, out-of-core solution for managing massive multi-dimensional array data. It excels at storing and accessing large and sparse datasets, making it an ideal choice for handling single-cell RNA sequencing data.

We utilized FlatBuffers to compress and quickly transfer large chunks of cell metadata and gene expressions to clients.

Categorical metadata is integer-encoded, and gene expressions are digitized to maximize compressibility.

To optimize the performance of differential expression, we engineered a custom implementation of Roaring Bitmaps, an efficient encoding method used for compressing postings lists, which contain cell indices in user requests. This algorithm and numerous other optimizations in the backend allow for the interactive comparison of populations of up to 1.5 million cells in under 60 s.

Finally, we built a custom renderer in WebGL, a JavaScript API for rendering high-performance graphics, to efficiently render millions of points in the cell embeddings and respond with minimal latency to user interaction.

### Data processing and normalization for Gene Expression

*Note that the following reflects the normalization approach and marker gene pipeline as of the time of writing (October 2023); as we are always seeking to improve our tools, please see the online documentation for any changes*.

#### Removal of duplicate cells

Some data on CZ CELLxGENE is duplicated due to independent submissions, for example, meta-analysis versus original data. All data submitted on Discover is curated to indicate whether any cell is the primary data. Only cells demarcated as primary data are included in the processing steps below.

#### Removal of low coverage cells

Any cell that has <500 genes expressed is excluded, which filters out about 8% of all data and does not eliminate any cell type in its entirety. This filter reduces noise in the gene expression estimates.

#### Removal of cells based on sequencing assay

Only cells from sequencing assays that measure gene expression and do not require gene-length normalization are included (Table 1).

**Table 1.** Sequencing assays included in Gene Expression normalized data object

Assay	EFO ontology term ID
sci-RNA-seq	EFO:0010550
10 × 3′ v1	EFO:0009901
10 × 5′ v1	EFO:0011025
10 × 3′ v2	EFO:0009899
10 × 5′ v2	EFO:0009900
10 × 3′ v3	EFO:0009922
10 × 3′ transcription profiling	EFO:0030003
10 × 5′ transcription profiling	EFO:0030004
10× technology	EFO:0008995
Seq-Well	EFO:0008919
Drop-seq	EFO:0008722
CEL-seq2	EFO:0010010

#### Data normalization

We chose to normalize raw counts from this dataset to log-transformed scaled pseudocounts (‘log transformation’) based on requirements that we established to meet a general use case of enabling scientists to explore gene expression across all eligible datasets in the corpus: it is a minimal, interpretable manipulation of the data; it is scalable to millions of cells; and it stabilizes the variance to a smaller range, suitable for visualization and rapid exploration.

Read counts are normalized using the ln (CPTT + 1) transformation of raw counts, where CPTT is Counts Per Ten Thousand.

Normalized matrices from multiple datasets of the same tissue are concatenated along the gene axis.

#### Removal of noisy ultra-low expression values

After applying normalization, any gene/cell combination counts less or equal than 3 are set to missing data. This allows for removal of noise due to ultra-lowly expressed genes and provides a cleaner visualization.

#### Summarization of data in heatmap

For each gene/cell type combination, the average ln (CPTT + 1)-normalized gene expression among genes that have a non-zero value is visualized by the dot color. The percentage of cells of any given cell type that express the gene of interest is visualized by the heatmap dot size, with the absolute number of cells expressing the gene found in parentheses. These numbers are calculated after the removal of low coverage cells.

#### Expression and cell count rollup across descendants in the Cell Ontology

The Cell Ontology is a hierarchical tree structure that represents the relationship between cell types. For example, the cell type ‘B cell’ is a descendant of the cell type ‘lymphocyte’. For a particular cell type, the Cell Ontology is used to sum up the expression values and cell counts of cells labeled as that cell type as well as those labeled as its descendants. In the aforementioned example, the average expression of ‘lymphocyte’ would include ‘B cells’ and all its other descendants.

This roll-up operation accounts for the fact that different datasets may have labeled their cells with different levels of granularity. It provides a more robust measure of the average expression of low-granularity cell type terms, such as ‘secretory cell’ or ‘lymphocyte’.

### Data-generated marker genes

For each of the cell types available in the data corpus, we use a Welch’s *t*-test on the normalized values to compare the average expression of each gene in the cell type of interest against each other cell type in the same tissue. For each gene, we can take the 10th percentile of effect sizes across all these cell type comparisons as the reported effect size. However, for small numbers of comparisons, the 10th percentile can be a noisy metric. To improve its robustness, we bootstrap the distribution of effect sizes, taking the 10th percentile of each replicate, and averaging all replicates to get the final effect size. We return the 25 genes with the top effect sizes.

It is important to note that some methodological decisions were made to balance accuracy with efficiency and scalability. For example, we use a *t*-test to perform differential expression, which is a simple and fast test. However, it may not be as accurate as more sophisticated (and computationally intensive) statistical tests. Differential expression values were calculated using normalized and log-transformed values instead of raw counts. Applying secondary filters to the data (like disease, ethnicity, etc.) does not update the returned marker genes; enabling dynamic calculation of marker genes for arbitrary populations of cells in arbitrary subsets of the data may be a direction for future development.

### Biological validation of CZ CELLxGENE normalization methods

1. Batch effects analyses

*Note that the batch effect analyses used a scaling factor of 1 000 000 – rather than 10 000 – for the log transformation of scaled pseudocounts normalization*.

ANOVA on average normalized gene expression values across covariates

We computed a one-way repeated measures ANOVA treating the average gene expression value of a particular gene as the dependent variable and the covariate as the independent variable. Average gene expression values were computed by excluding normalized gene expression values of 0. We looked at five different cell types and compared values across marker genes and housekeeping genes. Since there are genes that do not appear across all covariate values, the system design was unbalanced. Our null hypothesis H_0_ was that there is no significant difference between the average normalized gene expression values across covariate values. For most cell types, the *P* values obtained were >0.05, which means that we did not have sufficient evidence to reject the null hypothesis. We used the pingouin package to compute the scores. We looked at Dataset ID and Sequencing Assay as potential covariates.

2. Marker gene sensitivity

We selected 127 cell types across 14 tissues which had both canonical marker genes available in HuBMAP and raw count data available in CZ CELLxGENE: Gene Expression data corpus. We then normalized the raw counts for each tissue using either ln (CPTT + 1) or quantile normalization. Using these normalized values, we then computed marker genes for each of these cell types using the methods described above; however, we used a Student’s *t*-test instead of Welch’s simply based on the availability of this method in scanpy rather than requiring the full production pipeline for the validation study.

### TileDB-SOMA development for census

We worked in collaboration with TileDB to develop a technology for efficient and scalable single-cell data handling. Our efforts resulted in the abstract API specification, SOMA (‘Stack of Matrices, Annotated’) () and its Python and R implementations via TileDB-SOMA.

TileDB-SOMA was then used as the foundation to build Census for efficient programmatic access to CZ CELLxGENE data. SOMA provides an API and data model for single-cell data to store and access larger-than-memory datasets by providing query-ready data management for reading and writing at low latency and cloud scale.

The data model behind the SOMA specification is flexible and extensible, and it is inspired by existing single-cell data formats, notably AnnData. It can accommodate multiple measurements from derivative views (e.g. spatial and non-spatial data) and embeddings of sparse and dense data, along with both observation (e.g. cells) and feature (e.g. genes) axis annotations. Importantly the flexibility of the SOMA data model allows for representations beyond the single-dataset paradigm and enables managing single-cell data from multiple modalities (e.g. RNA, spatial, epigenomics) across joint or disjoint observations.

SOMA’s data model provides fundamental building blocks that can be composed into an arbitrary structure for a particular use case. These building blocks include: (i) Collections that act as containers for other SOMA data types; (ii) Experiments that are specialized collections to meaningfully group single-cell data measurements and allow for observation- and feature-based queries; (iii) Measurements that are specialized collections of single-cell data from a shared molecular measurement (e.g. RNA or protein); (iv) Data Frames that provide multi-column tables; and (v) Multi-dimensional Arrays (Sparse and Dense) to represent the multi-dimensional numeric data.

Leveraging this model, Census data is packaged into multiple Experiments, one per organism (*Homo sapiens* and *Mus musculus*, currently). Each experiment contains a Measurement for RNA data and an ‘obs’ Data Frame for cell metadata and annotations. The Measurement contains a ‘var’ Data Frame for gene metadata and a Collection of ‘X’ (expression data) layers. Currently, two layers are provided: one for RNA transcript raw counts and one for library-size normalized counts (Figure 6). Census data is packaged into multiple Experiments, one per organism (*H. sapiens* and *M. musculus*, currently). Each experiment contains a Measurement for RNA data and an ‘obs’ Data Frame for cell metadata and annotations. The Measurement contains a ‘var’ Data Frame for gene metadata and a Collection of ‘X’ (expression data) layers. Currently, two layers are provided: One for RNA transcript raw counts and one for library-size normalized counts. An additional top-level Collection of summary information Data Frames provide a profile of the contents of the Census data.

All query results are returned to the client via an iteration pattern that streams the data to the client incrementally, limiting the size of the data that must be handled at any given time to a fixed size ‘chunk’.

import CellxGene_census as cc

import tiledbsoma as soma

census = cc.open_soma ()

experiment = census[‘census_data’][‘homo_sapiens’]

axis_query = soma.AxisQuery (value_filter=‘tissue_general == ‘lung’’)

with experiment.axis_query (measurement_name=‘RNA’,

obs_query = axis_query) as query:

# Iterate over X data, returning ‘chunks’ of PyArrow tables

for table in query.X (‘raw’).tables ():

# PyArrow table can be converted to a Pandas DataFrame, e.g.

df = table.to_pandas ()

# Do something with df

print (df)

# Iterate over X data, returning ‘chunks’ of COO Matrices

for coo_matrix in query.X (‘raw’).coos ():

# Do something with coo matrix

print (coo_matrix)

In many cases, algorithms can be easily adapted to work with this incremental data access pattern. In particular, any computation that can operate independently on per-cell data should easily accommodate the SOMA API. However, computations that require random-access to the full query result or multiple passes through the data may need to be redesigned as an on-line algorithm. For example, computation of variance can be performed using Welford's online algorithm (71).

The SOMA specification is currently implemented by TileDB-SOMA, a Python and R client library that leverages TileDB (71) as its underlying database technology. By providing SOMA as a *specification* and TileDB-SOMA as one possible *implementation*, we aim to encourage the single-cell community to adopt the specification more broadly.

TileDB is an embedded database that enables a server-less architecture for accessing data stored in the cloud. TileDB’s support for cloud data storage means that it is highly optimized to minimize network latency and accomplishes this via use of indexing and compression techniques. As the Census is currently hosted on AWS S3, access to its data is effectively limited only by a user’s network bandwidth and compute resources. The TileDB querying engine supports efficient multi-dimensional slicing (i.e. fast reads), which naturally supports querying for Census data over the obs and var axes. CZI’s single-cell team had previously employed TileDB technology for other CZ CELLxGENE applications, including Explorer and Gene Expression.

## Supplementary Material

gkae1142_Supplemental_Files

## Data Availability

All source code for CZ CELLxGENE tools can be found at https://github.com/orgs/chanzuckerberg/repositories?&q=cellxgene. CZ CELLxGENE data can be accessed at https://CellxGene.cziscience.com/collections. Gene Expression data can be downloaded from the CZ CELLxGENE documentation site. The data and code used to assess the normalization methods behind CZ CELLxGENE: Gene Expression can be found at https://github.com/chanzuckerberg/cellxgene-manuscript-2023. For additional questions, please contact cellxgene@chanzuckerberg.com.
