# Supplementary material for: CZ CELLxGENE Discover: a single-cell data platform for scalable exploration, analysis and modeling of aggregated data
Source: Nucleic Acids Res. 2024 Nov 28;53(D1):D886–900. doi: 10.1093/nar/gkae1142 (PMC11701654; doi:10.1093/nar/gkae1142)
Supplement: gkae1142_Supplemental_Files [file gkae1142_supplemental_files.zip › Supplemental V3.pdf]

Supplemental Tables

Supplemental Table 1. CZ CELLxGENE documentation and resource links

**Description**	**Link**
CZ CELLxGENE Schema	https://github.com/chanzuckerberg/single-cell-curation/tree/main/schema
CZ CELLxGENE schema changelog	https://github.com/chanzuckerberg/single-cell-curation/blob/main/schema/5.2.0/schema.md#appendix-a-changelog
CZ CELLxGENE data eligibility criteria and submission process	https://CellxGene.cziscience.com/docs/032__Contribute%20and%20Publish%20Data
Gene Expression data processing & normalization documentation	https://CellxGene.cziscience.com/docs/04__Analyze%20Public%20Data/4_2__Gene%20Expression%20Documentation/4_2_3__Gene%20Expression%20Data%20Processing#data-normalization
Gene Expression source data	https://CellxGene.cziscience.com/docs/04__Analyze%20Public%20Data/4_2__Gene%20Expression%20Documentation/4_2_6__Gene%20Expression%20Source%20Data
TileDB-SOMA	https://github.com/single-cell-data/TileDB-SOMA
Census Python API	https://chanzuckerberg.github.io/CellxGene-census/python-api.html
Census data and schema	https://chanzuckerberg.github.io/CellxGene-census/CellxGene_census_docsite_schema.html#data-included-in-the-census

Supplemental Table 2. Metadata fields and corresponding ontologies

Metadata field	Ontology/Standards	Ontology link
organism (species)	NCBITaxon	https://obofoundry.org/ontology/ncbitaxon.html
development_stage (age)	HsapDv/MmusDv	https://obofoundry.org/ontology/hsapdv.html<br>https://obofoundry.org/ontology/mmusdv.html
is_primary_data	Boolean (true/false)	N/A
gene IDs	Ensembl	https://www.ebi.ac.uk/ols4/ontologies/ensemblglossary
sex	PATO	https://obofoundry.org/ontology/pato.html
self_reported_ethnicity	HANCESTRO	https://obofoundry.org/ontology/hancestro.html
disease	MONDO/PATO	https://obofoundry.org/ontology/mondo.html<br>https://obofoundry.org/ontology/pato.html
tissue	UBERON	https://obofoundry.org/ontology/uberon.html
suspension_type	[cell,nucleus]	N/A
assay	EFO	https://www.ebi.ac.uk/efo/
cell_type	CL	https://obofoundry.org/ontology/cl.html

Supplemental Figures

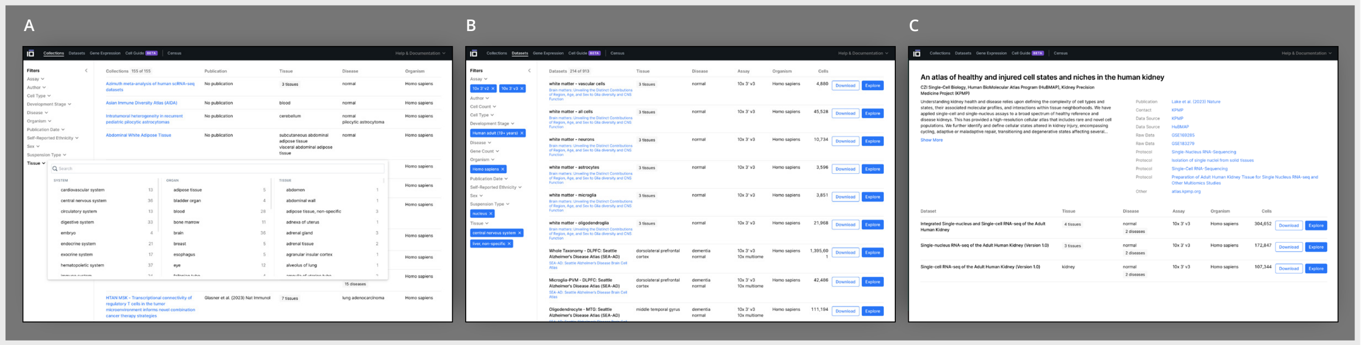

Figure S1. (A,B) CZ CellxGene User Interface (UI) allows for researchers to quickly filter and sort through datasets and collections based on collection metadata. (C) Each collection has an associated collections page with details about the author, study, datasets, and other collection metadata. From the collections page, researchers can easily explore any dataset that is part of the collection.

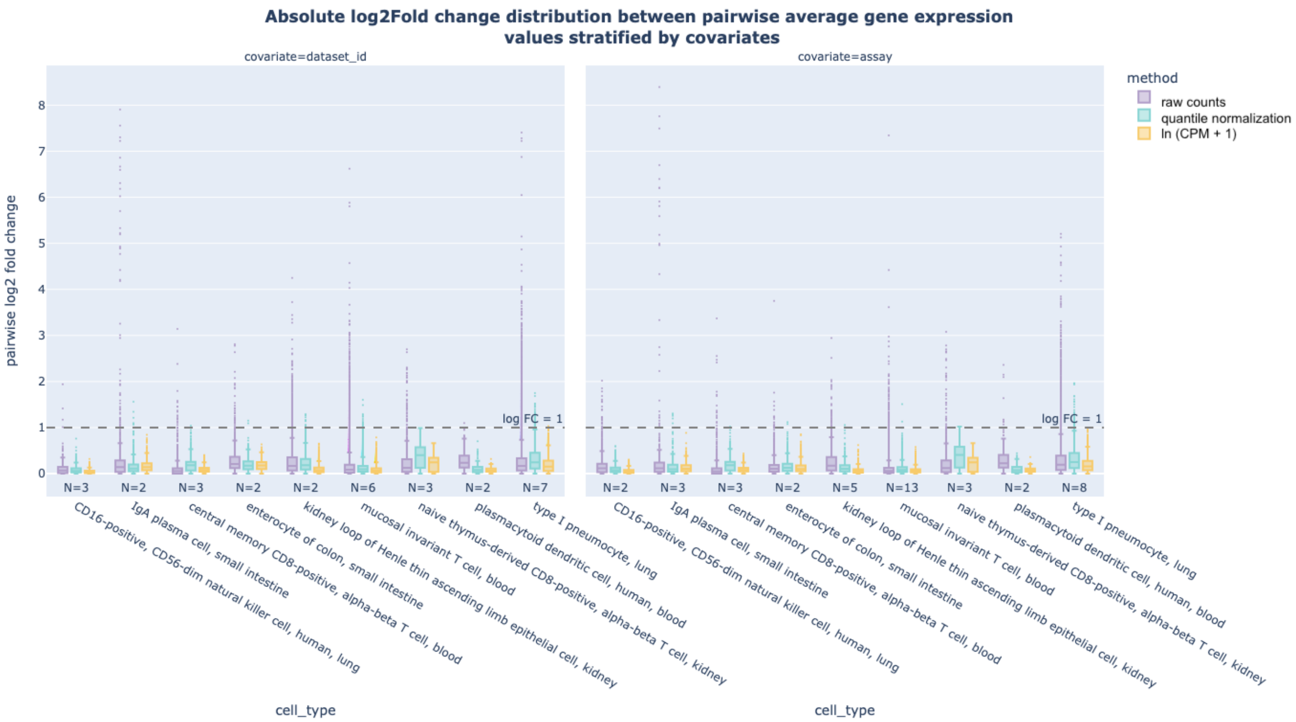

Figure S2. **Normalization further mitigates batch effects in average gene expression data.** Distribution of absolute log fold change values between pairwise average gene expression vectors stratified by covariates. Each point in the distribution represents the log fold change between the average gene expression values of a particular gene between two covariate values (e.g. one point in the Dataset ID covariate plot represents the log fold change between the average gene expression values of a particular gene in two different datasets). N is the number of covariate values (e.g. number of assays or datasets) per cell type. We consider all genes expressed in a particular cell type. Spread of log fold change between batches is highest for raw counts (σ_{raw} *dataset = 0.332*, σ_{raw} *assay* = 0.336), followed by quantile normalized values (σ_{qn} *dataset* = 0.170, σ_{qn} *assay* = 0.168), and log transform normalized values ($\sigma_{ln(CPM+1)}$dataset = 0.108, $\sigma_{ln\ (CPM+1)}$*assay* = 0.103).. Visualization is based on a random subsample of the data.

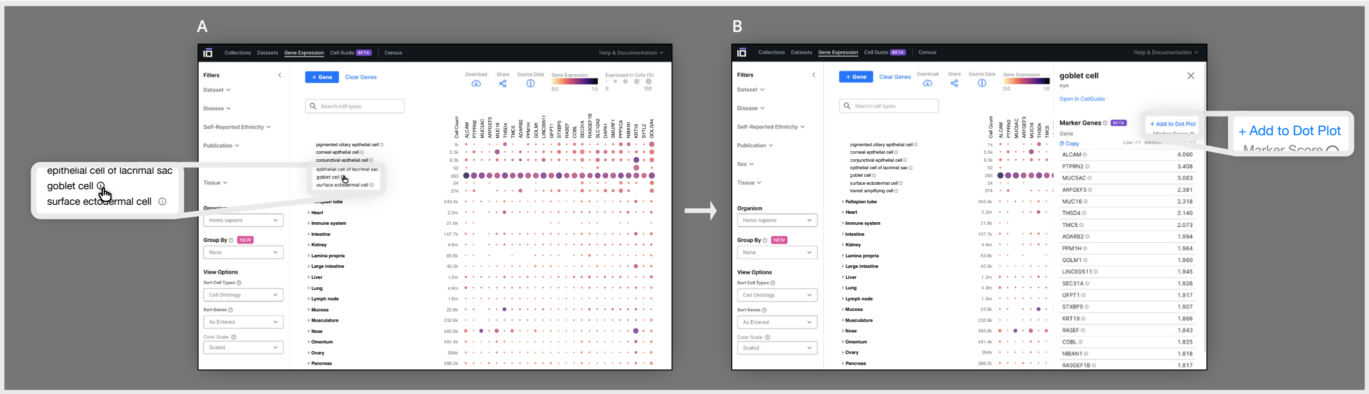

Figure S3. Visualizing marker genes across the data corpus. (A) From a Gene Expression heatmap generated from the log transform normalized data object,

researchers can identify maker genes for any given cell type present in the normalized data object by clicking on the info button next to the cell type name on the heatmap. For each cell, its top 25 marker genes (marker score > 0.5) are calculated using Welch's *t*-test to compare the gene expressions in the selected cell type to each other cell type in the tissue that is present in the data corpus. (B) All marker genes for a given cell type can be quickly added to the heat map by selecting Add to Dot Plot.

Supplemental Methods

Assessment of the impact of normalization on batch effects in aggregated gene expression data

If log transform normalization effectively mitigates batch effects in the averaged gene expression values, then we would expect to see similar average normalized values of gene expression between batches. To test this, we normalized the data, stratified by Dataset ID and Sequencing Assay values, and then computed the mean non-zero gene expression vectors for each covariate value (e.g., each dataset, each assay, etc). To assess the differences between these vectors, we calculated the log(fold change) between each pair, as reported in **Figure S2.**

More specifically: If a particular cell type has a total of n_k values for covariate *k*, we take the log fold changes between (n_k choose 2) pairs. For example, if a type I pneumocyte in lung is present across 8 different datasets, we are are computing the pairwise log fold changes between a total of (8 choose 2) = 28 pairs ($dataset_1/dataset_2$, $dataset_1/dataset_3$, …, $dataset_7/dataset_8$, etc). We take the absolute value of the log(fold change). Each pair of covariate values is only considered once (e.g. if the pair $dataset_1/dataset_2$ is included, the pair $dataset_2/dataset_1$ is not). We assessed dataset_id and assay as potential covariates. The reported standard deviations were calculated using the formula for average standard deviation for k groups of unequal size by taking the square root of the sum of variances of each individual cell type under a different covariate, weighted by the sample size divided by the total number of observations:

($\sigma_{cov,\ avg}$ = $_{(}$ct1-$1_{cov,ct12}$++ctn-$1_{cov,ctn2}$)/(total- κ))

where n_{ct1}, n_{ct2}, ..., n_{ctn} are the total number of samples for each cell type and $\sigma^2_{cov,ct}$ are the variances of these cell types in a particular covariate
