# Supplementary figures and images for: CZ CELLxGENE Discover: a single-cell data platform for scalable exploration, analysis and modeling of aggregated data
Source: Nucleic Acids Res. 2024 Nov 28;53(D1):D886–900. doi: 10.1093/nar/gkae1142 (PMC11701654; doi:10.1093/nar/gkae1142)

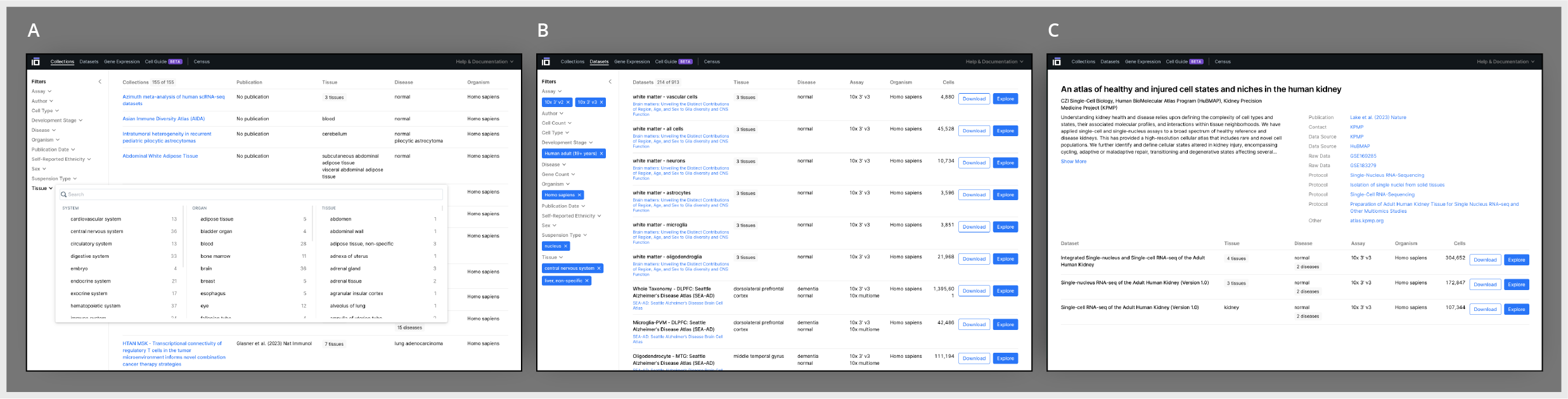

Supplement: gkae1142_Supplemental_Files [file gkae1142_supplemental_files.zip › 24_1025_CZ_CELLxGENE-Supplemental-Figure-1.png]

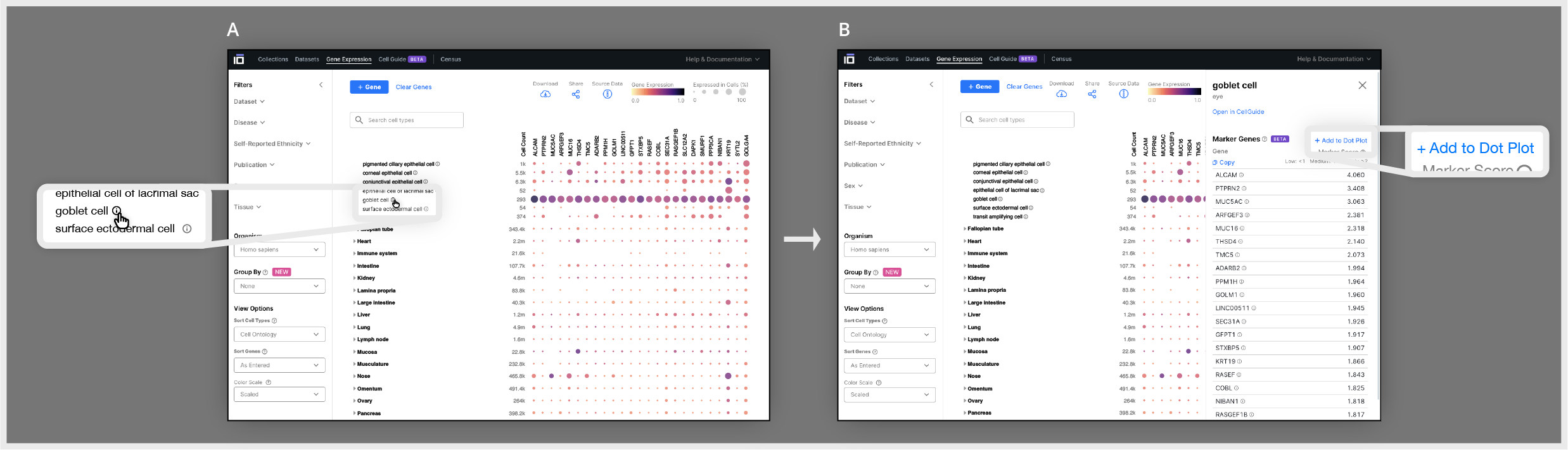

Supplement: gkae1142_Supplemental_Files [file gkae1142_supplemental_files.zip › 24_1025_CZ_CELLxGENE-Supplemental-Figure-3.png]

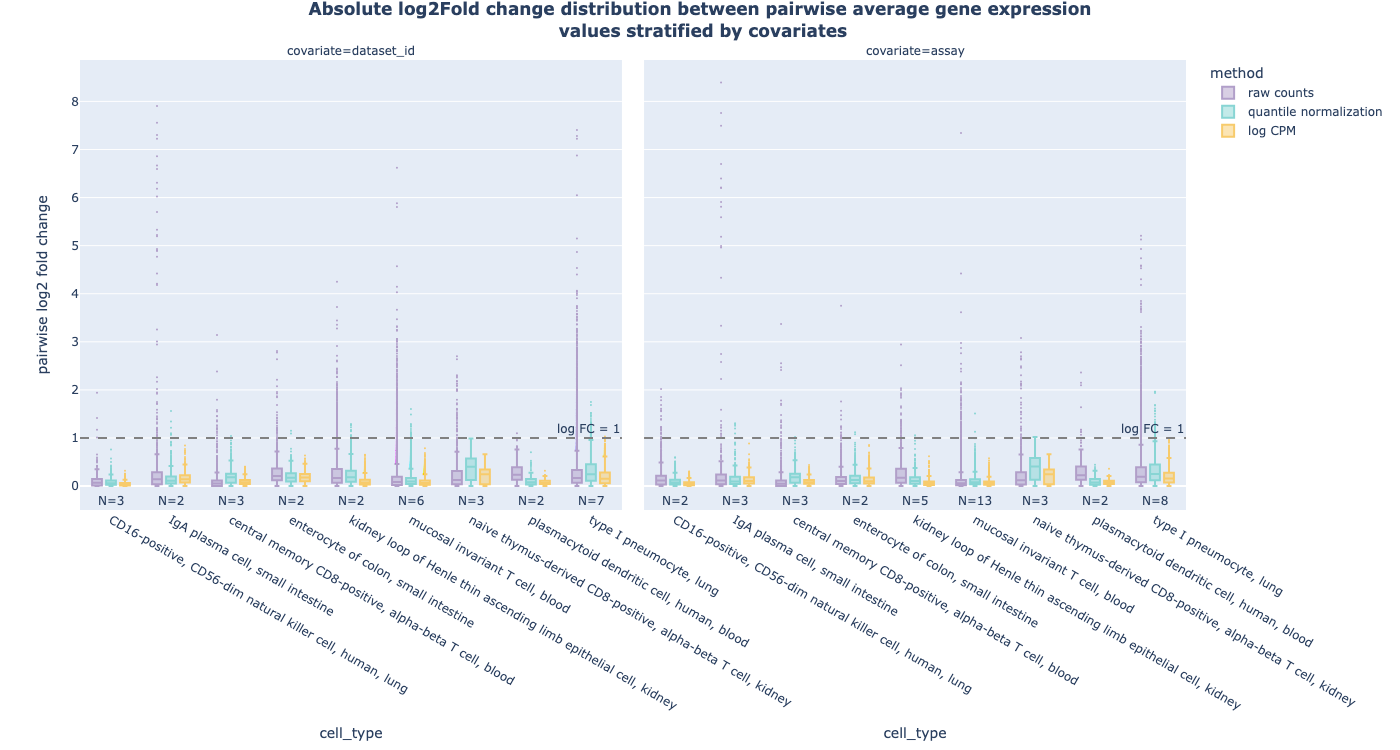

Supplement: gkae1142_Supplemental_Files [file gkae1142_supplemental_files.zip › Supplemental-Fig-2.png]
